# A Case of Hepatic Portal Venous Gas: Hypothesis of a Transient Direct Communication between a Penetrating Antral Gastric Ulcer and Mesenteric Varices

**DOI:** 10.1155/2017/8185132

**Published:** 2017-02-26

**Authors:** Hassan M. Ghoz, Shamlan M. Sheikh, Kanika Khandelwal, Joseph Fiore, Nicholas James, Joel Weinstock

**Affiliations:** ^1^Division of Internal Medicine, Steward Carney Hospital, Tufts School of Medicine, Dorchester, MA, USA; ^2^Division of Gastroenterology and Hepatology, Steward Carney Hospital, Tufts School of Medicine, Dorchester, MA, USA; ^3^Division of Pulmonology and Critical Care, Steward Carney Hospital, Tufts School of Medicine, Dorchester, MA, USA; ^4^Division of Gastroenterology and Hepatology, Tufts Medical Center, Tufts School of Medicine, Boston, MA, USA

## Abstract

Hepatic portal venous gas (HPVG) is a rare radiological sign that usually signifies an acute intra-abdominal process, most commonly bowel ischemia and sepsis. Few reports described an association with underlying gastric pathologies. We report a 60-year-old patient who presented with melena and chills and was discovered to have a gastric ulcer that appeared to have penetrated into a mesenteric varix. This, in turn, likely caused development of HPVG associated with fungemia. Treatment with a proton pump inhibitor and bowel rest was sufficient to resolve symptoms and the HPVG.

## 1. Background

Hepatic portal venous gas (HPVG) is a radiologic finding that usually denotes serious intra-abdominal pathology [1]. Intestinal ischemia and sepsis account for the majority of cases [2]. However, underlying gastric pathologies such as peptic ulcer disease and acute gastric dilation rarely can cause HPVG [3]. We are reporting a case of HPVG developing in a patient with mesenteric varices and a penetrating antral gastric ulcer. It is hypothesized that the ulcer penetrated a varix allowing introduction of gas into the portal system and fungemia.

## 2. Case Report

**Figure 1:**
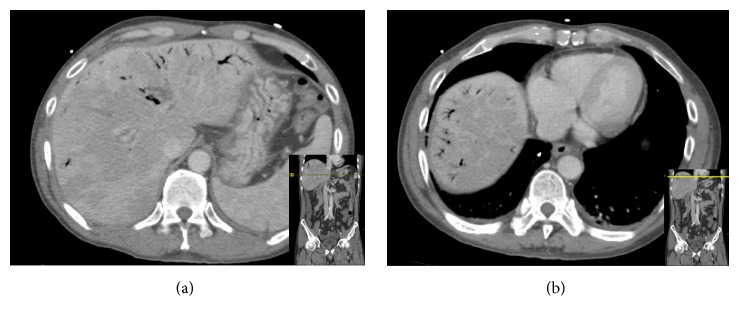
CT scan of the abdomen showing diffuse portal venous gas at different levels of the hepatic parenchyma.

**Figure 2:**
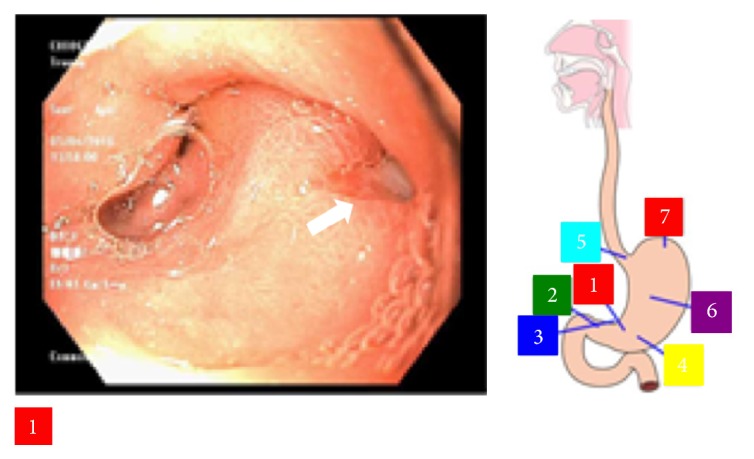
Endoscopic image showing the penetrating ulcer in the posterior wall of the gastric antrum.

**Figure 3:**
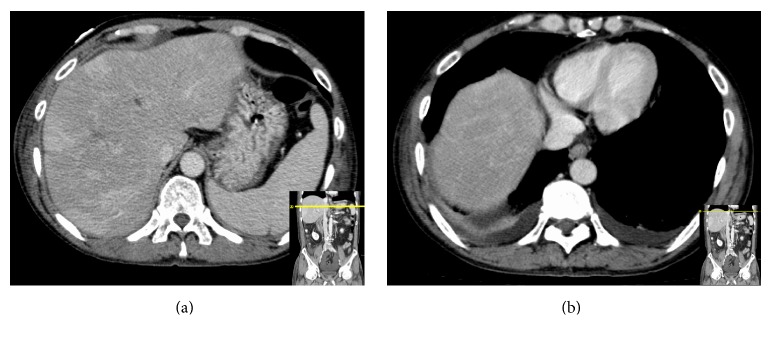
CT scan of the abdomen showing resolution of the portal venous gas.

**Figure 4:**
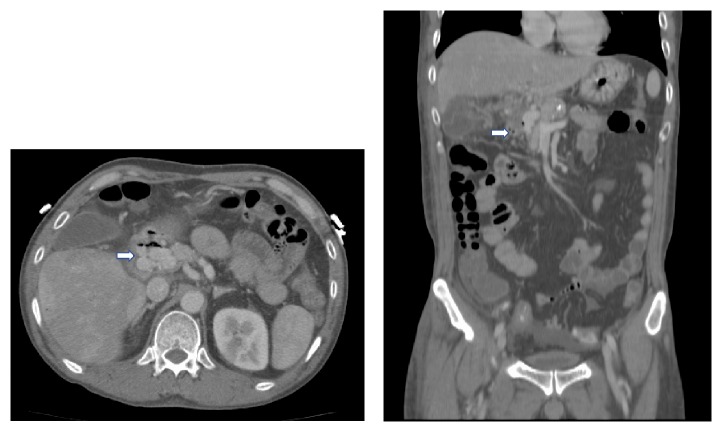
CT scan (coronal and axial views) showing multiple venous collaterals at the porta hepatis.

A 60-year-old Vietnamese male presented with malaise, fever, and chills for 2 days. Review of system revealed that he has been having dark loose stools for 2 days. The patient denied any abdominal pain. There was no significant past medical history and the patient was not taking any medications. The patient denied any recent NSAIDs use. He lived independently with his wife, smoked 0.5 pack-years of cigarettes for the past 20 years, and drinks a couple of beers on weekends. There was no history of any recreational drug use. On arrival to the ER, the patient was febrile (102.3 F), hypotensive (79/50 mmHg), and tachycardic (107 beats per minute). On physical exam, the patient appeared pale. All other general, cardiovascular, respiratory, abdominal, neurological, and extremity exams were benign. Stool guaiac was positive. His initial hemoglobin and hematocrit were 11.8 gm/dL and 34%, respectively, and remained stable during this hospitalization. White blood count and platelets levels were within the normal limit. His chemistries were significant for elevated aspartate aminotransferase (AST) of 55 U/L and alkaline phosphatase of 336 U/L. Alanine aminotransferase, lipase, lactic acid, creatinine, and blood urea nitrogen were normal. No recreational drugs were detected on urine toxicology screen. Nasogastric decompression through NG tube yielded 1 L of coffee ground fluid. Blood cultures were drawn. IV fluids and empiric antibiotics (levofloxacin and metronidazole) for possible intra-abdominal source were started as per sepsis protocol. The patient was admitted to the ICU for sepsis and upper GI bleed. He was also started on IV pantoprazole. CT scan of the abdomen/pelvis was done to perform an evaluation for possible intra-abdominal source. It showed extensive portal and mesenteric venous gas (Figures 1(a) and 1(b)). Also, it showed multiple collaterals at porta hepatis (Figure 4). Subsequently, an endoscopy was performed which revealed a normal esophagus and a 7 mm (largest diameter), nonbleeding, penetrating ulcer in the posterior wall of the gastric antrum (Figure 2). There was no evidence of gastric or esophageal varices. The patient was managed for upper gastrointestinal bleeding with intravenous proton pump inhibitor and bowel rest. A repeat CT scan performed 4 days later revealed interval resolution of previously visualized portal venous gas (Figures 3(a) and 3(b)). The patient's blood cultures yielded* Candida albicans* for which he was treated with antifungal therapy. Echocardiogram and eye examination were normal.* Helicobacter pylori* serum IgG antibody came back positive, and the patient was started on amoxicillin and clarithromycin. Other laboratory and serological panels (autoimmune, viral, tumor markers, ceruloplasmin, and iron levels) were within normal limits. Although the gastric ulcer was found to be nonbleeding, in the setting of multiple collaterals seen on CT abdomen, there was a concern of rebleeding. The patient was evaluated for a transjugular intrahepatic portosystemic shunt (TIPS) procedure to decrease the pressure in the portal system and thus decrease the likelihood of rebleeding from the mesenteric varices. His portal vein pressure was found to be only 16 mmHg, which did not indicate the need for a TIPS procedure. However, the pre-TIPS portal venogram showed acute thrombosis of mesenteric varices that were not amendable for intervening with coiling and also showed that the main portal vein was moderately stenotic from prior portal vein thrombosis. Furthermore, multiple small branches of the left and right portal veins were occluded with multiple enlarged collateral vessels. The patient was started on anticoagulation and discharged home 13 days after his admission.

## 3. Discussion

HPVG is a radiologic sign first described in 1955 by Wolfe and Evens in infants with necrotizing enterocolitis [4]. Since then, HPVG has been described in adults with bowel pathologies that disrupt the intestinal mucosa such as bowel ischemia, inflammatory bowel disease, and diverticulitis [5–7]. A proposed explanation for this association is that damage to the mucosal surface facilitates infiltration of intraluminal gas into the portal venous system [2]. In cases with intact mucosa, the presence of a gas forming organism should be suspected. Mechanical distention solely could disrupt the intestinal mucosal barriers offering a possible explanation to the association of bowel obstruction and paralytic ileus with HPVG [8, 9].

HPVG rarely is reported with gastric pathologies like perforated gastric ulcer, acute gastric dilation [3], and gastric cancer [8] and following gastric endoscopy and dilation [10]. There are few reported cases of HPVG developing with peptic ulcer disease [1, 2, 11].

Endoscopic examination of our patient revealed a nonbleeding, penetrating ulcer in the posterior wall of the gastric antrum secondary to* H. pylori* infection. We hypothesize that the penetrating peptic ulcer was the initial pathology and this could have led to a direct communication of the luminal contents with a mesenteric varix. Another possibility could be that venous bowel ischemia signified by the presence of chronic portal vein thrombosis with collaterals could have been present and led to the evolution of the portal venous gas as described by previous few reports [12, 13]. CT scan findings in addition to normal lactic acid levels, lack of significant gastrointestinal symptoms, and relatively benign abdominal examination excluded the more common intestinal pathologies leading to HPVG such as ischemia, diverticulitis, and inflammatory bowel disease.

HPVG is a sign that denotes a life-threatening condition but does not necessarily indicate a need for surgical intervention [11]. Management of HPVG is directed towards the underlying etiology. Resolution of HPVG is a sign of clinical improvement [14, 15]. In our case, the patient was managed with intravenous proton pump inhibitors for the gastric ulcer with complete bowel rest. Antibiotics initially were started for empiric coverage of the presumed intra-abdominal source of infection, which were switched to an antifungal agent once blood cultures grew* Candida albicans*.

We describe another case of HPVG that is associated with peptic ulcer disease in which we hypothesize that the ulcer might have allowed the entrance of luminal gas and fungus into an enlarged mesenteric varix. We also demonstrated the resolution of HPVG following treatment of the gastric ulcer with an intravenous proton pump inhibitor and bowel rest.
